# Using the net benefit regression framework to construct cost-effectiveness acceptability curves: an example using data from a trial of external loop recorders versus Holter monitoring for ambulatory monitoring of "community acquired" syncope

**DOI:** 10.1186/1472-6963-6-68

**Published:** 2006-06-06

**Authors:** Jeffrey S Hoch, Marie Antoinette Rockx, Andrew D Krahn

**Affiliations:** 1 Centre for Research on Inner City Health, St. Michael's Hospital, Toronto, Canada; 2 Department of Health Policy, Management and Evaluation, University of Toronto, Toronto, Canada; 3 Department of Medicine, University of Western Ontario, London Ontario, Canada

## Abstract

**Background:**

Cost-effectiveness acceptability curves (CEACs) describe the probability that a new treatment or intervention is cost-effective. The net benefit regression framework (NBRF) allows cost-effectiveness analysis to be done in a simple regression framework. The objective of the paper is to illustrate how net benefit regression can be used to construct a CEAC.

**Methods:**

One hundred patients referred for ambulatory monitoring with syncope or presyncope were randomized to a one-month external loop recorder (n = 49) or 48-hour Holter monitor (n = 51). The primary endpoint was symptom-rhythm correlation during monitoring. Direct costs were calculated based on the 2003 Ontario Health Insurance Plan (OHIP) fee schedule combined with hospital case costing of labour, materials, service and overhead costs for diagnostic testing and related equipment.

**Results:**

In the loop recorder group, 63.27% of patients (31/49) had symptom recurrence and successful activation, compared to 23.53% in the Holter group (12/51). The cost in US dollars for loop recording was $648.50 and $212.92 for Holter monitoring. The incremental cost-effectiveness ratio (ICER) of the loop recorder was $1,096 per extra successful diagnosis. The probability that the loop recorder was cost-effective compared to the Holter monitor was estimated using net benefit regression and plotted on a CEAC. In a sensitivity analysis, bootstrapping was used to examine the effect of distributional assumptions.

**Conclusion:**

The NBRF is straightforward to use and interpret. The resulting uncertainty surrounding the regression coefficient relates to the CEAC. When the link from the regression's p-value to the probability of cost-effectiveness is tentative, bootstrapping may be used.

## Background

Out patient ambulatory monitoring is often performed in patients with syncope (e.g., fainting or passing out) that present in the primary care setting to diagnose or exclude an arrhythmia, a potentially serious etiology [1-6]. This short-term monitoring device may take the form of an external loop recorder or a Holter monitor. The purpose of monitoring is to obtain a symptom-rhythm correlation during the monitored period (i.e., to have the monitoring device actively record a patient experiencing symptoms). Several studies have reported the diagnostic yield of the two monitoring modalities, suggesting a higher yield from the longer duration of monitoring provided by a loop recorder [3,7-12]. One recent randomized trial confirmed the higher diagnostic yield [5]. There is a lack of data about the cost of investigation of syncope presenting in the community. Referred and hospitalized patients are known to generate costs estimated between $3,000 and $25,000 dollars [13-19]. After a primary diagnostic trial [5], we sought to establish the cost of investigation of "community-acquired" syncope and to evaluate the cost-effectiveness of the two monitoring strategies in a prospective randomized trial [20].

A new health care treatment, intervention or technology is cost-effective if (1) the extra cost of (2) an extra unit of effect is less than (3) the decision maker's willingness to pay for it. A cost-effectiveness analysis (CEA) can report (1) and (2), representing two of the three pieces of information necessary to determine cost-effectiveness. Specifically, an incremental cost-effectiveness ratio (ICER) is the ratio of extra cost to extra effect (i.e., ΔC/ΔE). Thus, a CEA generates an estimate of the extra cost for an additional unit of effect, but the merit of the trade-off is typically a matter of opinion. In other words, the data are generally silent on whether the extra effect is worth the extra cost. For example, a new drug for multiple sclerosis may provide an extra quality adjusted life year (QALY) for £35,000. The new drug is cost-effective if the decision maker is willing to pay £35,000 or more for an extra QALY. Thus the verdict of cost-effectiveness depends upon the decision maker's willingness to pay (λ), a value not known from the cost and effect data. There is additional uncertainty beyond the fact that λ is unknown. The uncertainty comes from the fact that the sample ICER is a statistical estimate. For example, if the *true *ICER is £30,000 per QALY, the ICER *estimate *could be more or less due to sampling variability. In fact, the multiple sclerosis drug with the ICER estimate of £35,000 per QALY could have a true ICER of £30,000 per QALY. It would be a mistake to conclude there is no chance that the drug is cost-effective if λ = £31,000, for example.

The cost-effectiveness acceptability curve (CEAC) elegantly handles both uncertainty problems. This paper, building on recent work by Fenwick and colleagues [21], illustrates how to use the net benefit regression framework (NBRF) [22] to construct a CEAC. After a brief summary of relevant statistical concepts, this paper uses clinical trial data from a recently published CEA comparing external loop recorders with Holter monitors for ambulatory monitoring of syncope.

## Methods

One hundred patients referred for ambulatory monitoring with syncope or presyncope (hereafter described as syncope) were randomized to a one-month external loop recorder (n = 49) or 48-hour Holter monitor (n = 51). Patients provided written informed consent, and the protocol was approved by the University of Western Ontario Ethics Review Board. The primary endpoint was symptom-rhythm correlation during monitoring. Direct costs in Canadian dollars were calculated from the Ministry of Health's perspective based on the 2003 Ontario Health Insurance Plan (OHIP) fee schedule for professional fees and on hospital case costing data for the calculation of labour, materials, service and overhead for diagnostic testing and related equipment. Costs were converted to US Dollars using a conversion rate converted on July 20th, 2005 of ($1 USD = $1.21543 CAD) [20].

Loop recorders were both more costly and more effective than Holter monitors. For the loop recorder, the cost in US dollars was $648.50 and for the Holter monitor $212.92 [20]. The extra cost of $435.58 for the loop recorder was accompanied by a 39.74% increase of success while monitoring (in the loop recorder group 31 of 49 or 63.27% of patients had symptom recurrence and successful activation, compared to 12 of 51 or 23.53% in the Holter group). The ICER estimate was $1096 per additional diagnosis. The CEAC finds purchase here as there is uncertainty about the maximum a decision maker would pay for an additional diagnosis coupled with the statistical variability inherent in trial data. As an alternative to the method illustrated by Fenwick and colleagues [21], we use the NBRF to show how to construct the CEAC.

The CEAC has been advocated for summarizing the results of a CEA because it highlights the relationship between the assessment of cost-effectiveness and the unknown λ [23-27]. As originally described, the CEAC originates from a Bayesian context; however, the CEAC can be given a frequentist interpretation. For a given λ, the CEAC is equal to one minus the *one-sided *significance level for testing the null hypothesis that the "new treatment" is not cost-effective (i.e., the additional benefits are outweighed by the additional costs) [25,28]. Under this frequentist framework, the CEAC can be viewed as illustrating a decision rule for rejecting the null hypothesis that the intervention is not cost-effective.

Alternatively, the CEAC can be interpreted in a 'Bayesian' fashion [23,24] as: the probability that an individual, with a set of prior beliefs about the cost-effectiveness of the new treatment, now believes the new treatment to be cost-effective (i.e., the additional benefits outweigh the additional costs). While a Bayesian approach provides a well-justified interpretation for a CEAC, it presents other dilemmas. For example, there exist many 'Bayesian' CEACs – namely one for every set of prior beliefs – with no criteria for choosing between them. This is important because every CEAC is 'correct' for its given prior. Thus, the calculation of *a *Bayesian CEAC requires the specification of the prior distribution of the cost-effectiveness data before the data were collected. Typically as a reference case scenario, it is common and convenient to use a 'non-informative' prior which allows the data to overwhelm prior beliefs. However, except in the simplest of examples there is no agreement about the definition of a reference prior distribution and many so-called non-informative priors are not non-informative at all (see section 5.5.1 of [29]). When using a 'non-informative' prior with the NBRF (in this case assuming there is no reason to modify the results of the data analysis), the Bayesian mechanics work in the background and formal derivation of the posterior distribution can be avoided. In other words, one can run a net benefit regression and use the resulting parts to illustrate the probability that a new treatment or intervention is cost-effective (NB: The p-value itself does not provide an estimate of the probability of cost-effectiveness when there is prior information. This is a fundamental distinction between the interpretation of a p-value and a posterior probability [30]. For a more comprehensive discussion about the use of genuine prior information in cost-effectiveness analyses readers are referred to [31-33]).

The NBRF was introduced to facilitate the use of regression tools in economic evaluation [22]. Net benefit regression uses as the dependent variable, net benefit *nb*_*i *_= λ·*effect*_*i *_- *cost*_*i *_from person-level effect (*effect*_*i*_) and cost (*cost*_*i*_) data (as a matter of preference, the analyst may use net health benefits [34] instead of net monetary benefits [35]). When ordinary least squares (OLS) is used to estimate the simple linear regression

*nb*_*i *_= β_0 _+ β_1_*TX *+ ε

where *TX *is a "new treatment" indicator variable (e.g., *TX *= 1 if the patient received a loop recorder and *TX *= 0 if the patient received a Holter monitor), the coefficient estimate of β_1_, call this b_1_, equals the difference in mean *nb *for the loop and Holter groups. It can be shown [22] that when this difference is greater than zero (i.e., when the loop group has greater mean net benefits than the Holter group), then ΔC/ΔE < λ. In other words, if b_1 _> 0, then the loop recorder is cost-effective relative to the Holter monitor (or the incremental net benefit is positive). The statistical uncertainty involving the cost and effect data is expressed in the p-value for b_1_. The p-value for b_1 _can be used to make the y-axis of the CEAC [22,25]; however, caution must be exercised in two regards.

**Figure 1:**
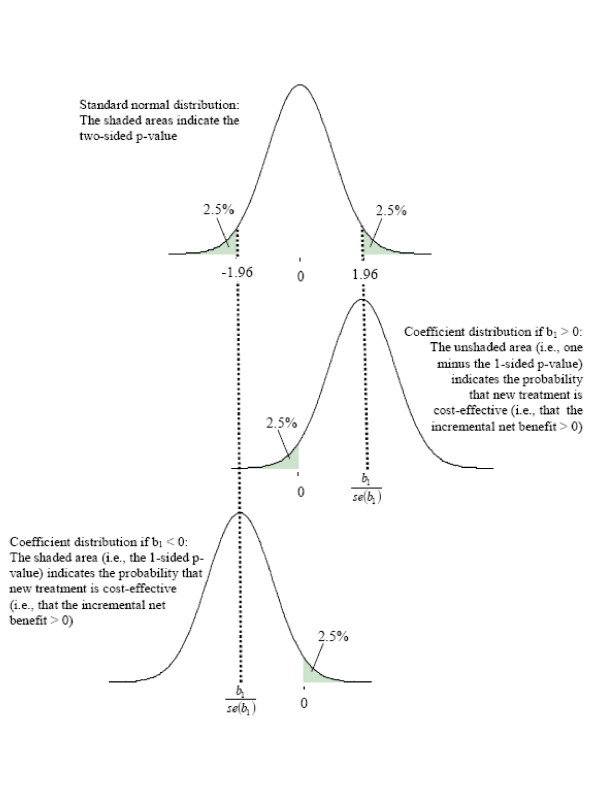
Illustrating the relationship between the p-value for the "new treatment" indicator variable in a net benefit regression (i.e., b_1_the incremental net benefit) and the probability that a new treatment is cost-effective

Using the NBRF and a Bayesian perspective, the CEAC illustrates the probability that a "new treatment" is cost-effective by graphing the probability that β_1 _> 0 as a function of λ. Most statistical packages have regression programs that report a two-sided p-value, but in this case a one-sided probability is indicated. Because the two-sided p-value is twice as much probability as is needed, it is necessary to divide it by two (this converts the two-sided p-value into a one-sided p-value). Figure 1 illustrates this and the importance of checking the sign of b_1_. When b_1 _< 0, the probability that new treatment is cost-effective equals the one-sided p-value, and when b_1 _> 0, the probability that new treatment is cost-effective equals one minus the one-sided p-value. Thus when using the p-value from a regression to make a CEAC, one must check that one is using the one-sided p-value and that one is doing the correct calculation given the sign of b_1 _(i.e., 1/2 p-value of b_1 _when b_1 _< 0 or 1 - 1/2 p-value of b_1 _when b_1 _> 0). Lastly, because the p-value of a parametric analysis is derived from a distributional assumption, non-parametric methods like bootstrapping may offer better alternatives when distributional concerns arise (e.g., the data do not appear distributed normally or with constant variance).

## Results

Each study participant who received a loop recorder incurred costs of $648.50 and 31 of the 49 (63.27%) had symptom recurrence and successful activation. In comparison, the Holter monitors cost $212.92 for each study participant and only 12 of the 51 (23.53%) experienced a successful outcome. The NBRF was implemented by estimating with OLS the regression

*nb*_*i *_= β_0 _+ β_1_*LOOP *+ ε

where *LOOP *is an indicator variable equaling one if the patient received a loop recorder and zero if the patient received a Holter monitor. Table 1 shows how the net benefit statistic (*nb*_*i*_) was calculated for each person when λ was set to $1000.

**Figure 2:**
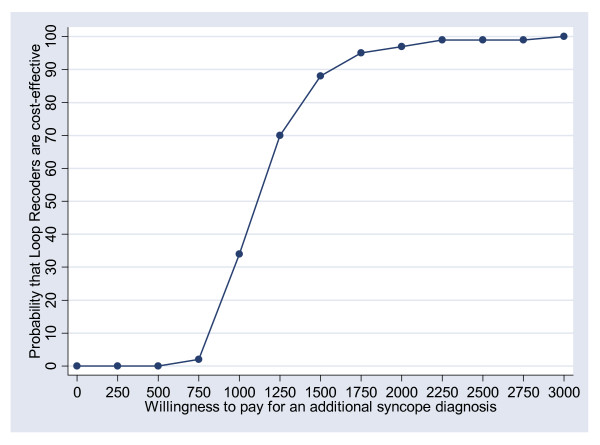
Cost-effectiveness acceptability curve (CEAC) showing the probability that loop recorders are cost-effective compared to Holter monitors over a range of values for willingness to pay for an additional syncope diagnosis.

Table 2 presents the complete results of five net benefit regressions using λ = $500, $1000, $1500, $2000 and $2500. To illustrate how the CEAC can be computed using net benefit regression, Table 3 lists regression estimates of the *LOOP *indicator variable for λ = $500 through $3000 (the horizontal axis for the CEAC) as well as the regression and bootstrap estimates of the probability that the loop recorder is cost-effective (the vertical axis for the CEAC). For λ < $1000, the estimate of the incremental net benefit is negative (i.e., b_1 _< 0), so the quantity to calculate for the CEAC's vertical axis is simply one half of the two-sided p-value. For λ > $1000, the estimate of the incremental net benefit is positive (i.e., b_1 _> 0), so the quantity to calculate for the CEAC's vertical axis is one minus one half of the two-sided p-value. Figure 2 illustrates the resulting CEAC. Because the p-value is based on distributional assumptions that may not hold with small sample sizes or non-constant variance, bootstrapping can be used as a non-parametric alternative to obtain values for the CEAC's vertical axis. For this example, we drew 1000 bootstrapped samples of n = 100 from our original sample. The resulting estimates of the probability that the incremental net benefit is positive are reported in the last column of Table 3.

## Discussion

A CEAC indicates a 50% chance of cost-effectiveness when λ equals the sample estimate of the ICER [26]. The ICER for the loop recorder was $1,096 per extra successful diagnosis. Table 3 shows that when λ is within $500 of the ICER estimate, the probability of cost-effectiveness is quite sensitive. For example, at λ = $500, the probability that loop recorders are cost-effective is 0%, but at λ = $1500 it is approximately 88%. Figure 2 illustrates this, as the most dramatic gains in the height of the curve (from 0% to 88%) occur between λ = $500 and $1500. Alternatively, the curve is mostly flat for λ < $ 500 and λ > $1500. While we may never know the real value of λ, if it is assumed to be near the low range of the costs generated by referred and hospitalized patients (e.g., $3000 dollars), there appears to be a good chance that loop recorders are cost-effective.

As reflected in the last two columns of Table 3, the probability of cost-effectiveness calculated using the p-value was nearly identical to that calculated using the bootstrapping method. This finding may be related to the fact that the cost data in this trial did not vary by patient within treatment group. All patients receiving a loop recorder had costs of $648.50 and all patients receiving a Holter monitor had costs of $212.92. When both patient level costs and effect data vary, net benefit regression can still be used to construct a CEAC (i.e., the statistical uncertainty involving the cost and effect data is expressed in the p-value). However, the assumptions necessary to use the p-value may not hold; for example, the presence of skewness or heteroskedasticity in the data suggests caution when using the p-value. Indeed, for low values of λ, the almost inevitable non-normal distribution of costs can challenge the assumptions made in using the p-value in the regression approach. For this reason, empirical examples of the NBRF typically use bootstrapping to generate CEACs [36-38]. However, as noted by a reviewer, the bootstrap is not necessarily robust, particularly in CEAs when there is also concern about the use of parametric methods because of skewness. In addition to the incremental net benefit (β_1_), net benefit regression provides an estimate of the mean net benefit of "usual care" (β_0_), the mean net benefit of "new treatment" (β_0 _+ β_1_) and also regression diagnostic information (e.g., the residual errors and R^2^). Thus, the NBRF facilitates using regression diagnostics (see the "Regression Diagnostics" section and Figure 6 in [22]) to improve the quality of economic evaluations.

## Conclusion

The NBRF provides a way for economic evaluations to use the variety of tools that have been developed for regression. For any value of λ, net benefit regression produces a cost-effectiveness estimate, and the CEAC produces a cost-effectiveness probability. To allow for the fact that the analyst does not know the decision maker's λ, the horizontal axis of a CEAC varies in the style of a sensitivity analysis, and the statistical uncertainty about cost-effectiveness is reflected on the vertical axis. This paper has illustrated how the NBRF can be used to construct a CEAC. When the link from a net benefit regression's p-value to the probability of cost-effectiveness is tentative, bootstrapping provides an alternative.

## Abbreviations

CEACs: cost-effectiveness acceptability curves

ICER: incrememtnal cost-effectiveness ratio

NBRF: net benefit regression framework

OHIP: Ontario Health Insurance Plan

OLS: ordinary least squares

QALY: quality adjusted life year

## Competing interests

The author(s) declare that they have no competing interests.

## Authors' contributions

JSH- 1) made substantial contributions to conception and design, analysis and interpretation of data; 2) was involved in drafting the manuscript and revising the manuscript critically for important intellectual content; and 3) has given final approval of the version to be published.

MAR- 1) made substantial contributions to the conception and design, analysis and interpretation of data; 2) was involved in revising the manuscript critically for important intellectual content; and 3) has given final approval of the version to be published.

ADK- 1) made substantial contributions to conception and design, analysis and interpretation of data; 2) was involved in revising the manuscript critically for important intellectual content; and 3) has given final approval of the version to be published.

The first Figure in *bmcFIGS9.doc *is Figure 1 (it is formatted to fit portrait). The second figure in *bmcFIGS9.doc *is Figure 2 (it is formatted to fit landscape).

## Pre-publication history

The pre-publication history for this paper can be accessed here:



## Figures and Tables

**Table 1:** Construction of the dependent variable (net benefit) when λ = $1000

Net Benefit with λ = $1000	Number of Subjects	Treatment Group	Successful outcome
$1000 * 1 - $648.50 = $351.50	31	Loop recorder	Yes
$1000 * 0 - $648.50 = - $648.50	18	Loop recorder	No
$1000 * 1 - $212.92 = $787.08	12	Holter monitor	Yes
$1000 * 0 - $212.92 = - $212.92	39	Holter monitor	No

NOTE: All loop recorders cost $648.50 and all Holter monitors cost $212.92.

**Table 2:** Simple net benefit regression estimates (N = 100)^a^

Explanatory variables	λ = $500^b ^coefficient (p-value)	λ = $1000 coefficient (p-value)	λ = $1500 coefficient (p-value)	λ = $2000 coefficient (p-value)	λ = $2500 coefficient (p-value)
Constant term	-95.27 (0.004)	22.37 (0.728)	140.02 (0.149)	257.67 (0.047)	375.32 (0.021)
*LOOP*	-236.90 (<0.001)	-38.22 (0.678)	160.46 (0.246)	359.14 (0.053)	557.82 (0.017)
					
R-squared	0.2143	0.0018	0.0137	0.0377	0.0570

^a ^The treatment indicator variable *LOOP *= 1 for the Loop recorder and 0 for the Holter monitor.

^b ^All monetary figures are in US dollars.

**Table 3:** Using the net benefit regression results to create a cost-effectiveness acceptability curve (CEAC) with a comparison to bootstrapping the probability of cost-effectiveness

λ	Treatment Indicator Coefficient		One sided p-value	Probability of cost-effectiveness (regression)	Probability of cost-effectiveness (bootstrapping)
				
	Estimate	p-value			
$500	-236.90	<0.001	≈ 0.000	0%	0%
$750	-137.56	0.048	0.024	2%	2%
$1000	-38.22	0.678	0.339	34%	33%
$1250	61.12	0.595	0.298	70%	71%
$1500	160.46	0.246	0.123	88%	89%
$1750	259.80	0.108	0.054	95%	94%
$2000	359.14	0.053	0.027	97%	97%
$2250	458.48	0.028	0.014	99%	98%
$2500	557.82	0.017	0.009	99%	99%
$2750	657.16	0.011	0.006	99%	99%
$3000	756.50	0.007	0.004	100%	100%
